# Supplementary material for: Diet modulates the therapeutic effects of dimethyl fumarate mediated by the immunometabolic neutrophil receptor HCAR2
Source: eLife. 2025 Apr 23;14:e98970. doi: 10.7554/eLife.98970 (PMC12113270; doi:10.7554/eLife.98970)
Supplement: Supplementary file 1. [file elife-98970-supp1.docx]

**Supplementary File 1**. Main ingredients in the diets.

| **Ingredients** | **Normal chow diet, Catalogue number 1314M Altromin** | **High fibre diet, Catalogue number C1000 modified, #100213 Altromin** | **Lauric acid diet, Catalogue number C1000 modified, #100212 Altromin** |
| --- | --- | --- | --- |
| Crude Protein | 22.5% | 12% | 20% |
| Crude Fat | 5.1% | 5% | 30% |
| Crude Fiber | 4.5% | 35% | 5% |
| Polysaccharides | 35% | 19% | 23% |
| Metabolizable Energy | 3,339 kcal/kg | 1,894 kcal/kg | 4,748 kcal/kg |
| Capric acid C-10:0 |  |  | 1.8% |
| Lauric acid C-12:0 |  | 0.0% | 13.2% |
| Myristic acid C-14:0 |  | 0.0% | 5.7% |
| Palmitic acid C-16:0 | 0.5% | 0.2% | 3.3% |
| Stearic acid C-18:0 | 0.2% | 0.1% | 2.2% |
| Oleic acid C-18:1 | 0.9% | 1.2% | 1.6% |
| Linoleic acid C-18:2 | 2.2% | 2.5% | 1.6% |
