# Supplementary material for: Diet modulates the therapeutic effects of dimethyl fumarate mediated by the immunometabolic neutrophil receptor HCAR2
Source: eLife. 2025 Apr 23;14:e98970. doi: 10.7554/eLife.98970 (PMC12113270; doi:10.7554/eLife.98970)
Supplement: Supplementary file 5. [file elife-98970-supp5.docx]

**Supplementary File 5.** Antibodies used for flow cytometry.

| **Antibody name** | **Final**  **Concentration** | **Catalog number** | **Supplier** |
| --- | --- | --- | --- |
| Brilliant Violet 650 anti-mouse/human CD11b | 2 µg/ml | 101239 | BioLegend |
| Brilliant Violet 510 anti-mouse CD45 | 2 µg/ml | 103138 | BioLegend |
| PerCP/Cyanine 5.5 anti-mouse Ly-6G | 2 µg/ml | 127616 | BioLegend |
| PE/Cyanine 7 anti-mouse Ly-6C | 0.1 µg/ml | 128018 | BioLegend |
| FITC anti-mouse CD3ɛ | 2 µg/ml | 100326 | BioLegend |
