# Supplementary material for: Diet modulates the therapeutic effects of dimethyl fumarate mediated by the immunometabolic neutrophil receptor HCAR2
Source: eLife. 2025 Apr 23;14:e98970. doi: 10.7554/eLife.98970 (PMC12113270; doi:10.7554/eLife.98970)
Supplement: Supplementary file 6. — f, female; m, male. [file elife-98970-supp6.docx]

**Supplementary File 6**. Results of statistical analyses. f, female; m, male.

| **Figure** | **Sample size (n)** | **Statistical test** | **Values** |
| --- | --- | --- | --- |
| Fig. 1B | 10 (f)  mice/group | Two-way ANOVA for AUC with Sidak posthoc test | diet: F (2, 54) = 81.3, *p < 0.0001*  treatment: F (1, 54) = 39.89, *p < 0.0001*  interaction: F (2, 54) = 149.5, p < 0.0001  LAD veh vs. LAD DMF:  *p < 0.0001*  NCD veh vs. NCD DMF:  *p < 0.0001*  HFbD veh vs. HFbD DMF:  *p < 0.0001* |
| Fig. 1D, AUC | 10 (f)  mice/group | Scheirer-Ray-Hare test followed by targeted Mann-Whitney U test; Bonferroni-Holm corrected | diet: *χ²* (2) = 6.39, *p = 0.0268*  treatment: *χ²* (1) = 6.44, *p = 0.041*  interaction: χ² (2) = 7.24, *p = 0.0112*  HFbD DMF vs. HFbD veh: *p = 0.0148*  NCD DMF vs. LAD DMF: *p = 0.0084*  HFbD DMF vs. LAD DMF: *p = 0.0095* |
| Fig. 1D, Neuroscore | 10 (f)  mice/group | Scheirer-Ray-Hare test followed by targeted Mann-Whitney U test; Bonferroni-Holm corrected | diet: *χ²* (2) = 7.55, *p = 0.0229*  treatment: *χ²* (1) = 4.74, *p = 0.0295*  interaction: χ² (2) = 8.97, *p = 0.0113*  HFbD DMF vs. HFbD veh: *p = 0.0092*  NCD DMF vs. LAD DMF: *p = 0.005*  HFbD DMF vs. LAD DMF: *p = 0.0048* |
| Fig. 1D, Onset | 10 (f)  mice/group | Scheirer-Ray-Hare test followed by targeted Mann-Whitney U test; Bonferroni-Holm corrected | diet: *χ²* (2) = 4.11, *p = 0.1281*  treatment: *χ²* (1) = 13.12, *p < 0.0003*  interaction: χ² (2) = 6.66, *p = 0.0358*  HFbD DMF vs. HFbD veh: *p = 0.0012*  NCD DMF vs. LAD DMF: *p = 0.006*  HFbD DMF vs. LAD DMF: *p = 0.006* |
| Fig. 2A | 9-10 (f) mice/group | One-way ANOVA | *F* (2, 26) = 22.73,  *p = 0.0839* |
| Fig. 2C | 9-10 (f) mice/group | Mann-Whitney U test, FDR adjusted | *p* values for individual lipids are listed in Supplementary Table 2 |
| Fig. 2D | 9-10 (f) mice/group | Kruskal-Wallis test with Dunn’s posthoc test | LAD vs. NCD: *p* = 0.0086  LAD vs. HFbD: *p* = 0.0001 |
| Fig. 2E | 8-10 (f) mice/group | Mann-Whitney U test | *p* values for individual lipids are listed in Supplementary Table 3 |
| Figure 2 – figure supplement 1A, Acetic Acid | 9-10 (f) mice/group | Two – way ANOVA | diet: *F* (2, 52) = 2.59,  *p* = *0.0842*  treatment: *F* (1,52) = 0.7765, *p* = *0.3823*  interaction: *F* (2,52) = 1.21,  *p* = *0.3074* |
| Figure 2 – figure supplement 1B, Propionic Acid | 9-10 (f) mice/group | Two – way ANOVA | diet: *F* (2, 52) = 0.05,  *p* = *0.9512*  treatment: *F* (1,52) = 0.6346, *p* = *0.4293*  interaction: *F* (2,52) = 0.40,  *p* = *0.6708* |
| Fig. 3B, Glycitein | 8-10 (f) mice/group | Scheirer-Ray-Hare test followed by targeted Mann-Whitney U test; Bonferroni-Holm corrected | diet: *χ²* (2) = 42.94, *p < 0.0001*  *treatment: χ²* (1) = 0.07, *p = 0.7913*  interaction: χ² (2) = 0.32, *p = 0.8521*  NCD DMF vs. LAD DMF: *p < 0.001*  HFbD DMF vs. NCD DMF: *p < 0.001*  NCD veh vs. LAD veh: *p < 0.001*  HFbD veh vs. LAD veh: *p = 0.0082*  HFbD veh vs. NCD veh: *p < 0.001* |
| Fig. 3B, Equol | 8-10 (f) mice/group | Scheirer-Ray-Hare test followed by targeted Mann-Whitney U test; Bonferroni-Holm corrected | diet: *χ²* (2) = 40.77, *p < 0.0001*  *treatment: χ²* (1) = 0.01, *p = 0.9203*  interaction: χ² (2) = 1.54, *p = 0.4630*  NCD DMF vs. LAD DMF: *p < 0.001*  HFbD DMF vs. NCD DMF: *p < 0.001*  NCD veh vs. LAD veh: *p < 0.001*  HFbD veh vs. LAD veh: *p = 0.0105*  HFbD veh vs. NCD veh: *p < 0.001* |
| Fig. 3C,  N-Acetyltyrosine | 8-10 (f) mice/group | Scheirer-Ray-Hare test followed by targeted Mann-Whitney U test; Bonferroni-Holm corrected | diet: *χ²* (2) = 15.69, *p = 0.0004*  treatment: *χ²* (1) = 0.07, *p = 0.7913*  interaction: χ² (2) = 0.98, *p = 0.6126*  HFbD veh vs. LAD veh: *p = 0.0246* |
| Fig. 3C,  sphingosine 1-phosphate | 8-10 (f) mice/group | Scheirer-Ray-Hare test followed by targeted Mann-Whitney U test; Bonferroni-Holm corrected | diet: *χ²* (2) = 14.85, *p = 0.0006*  treatment: *χ²* (1) = 0.80, *p = 0.3711*  interaction: χ² (2) = 2.74, *p = 0.2541*  NCD DMF vs. LAD DMF: *p = 0.031*  HFbD DMF vs. LAD DMF: *p = 0.0126* |
| Fig. 3D,  Glutamyl-glutamine | 8-10 (f) mice/group | Scheirer-Ray-Hare test followed by targeted Mann-Whitney U test; Bonferroni-Holm corrected | diet: *χ²* (2) = 4.13, *p = 0.1268*  treatment: *χ²* (1) = 22.45, *p < 0.0001*  interaction: χ² (2) = 3.08, *p = 0.2144*  LAD DMF vs. LAD veh: *p = 0.002*  HFbD DMF vs. HFbD veh: *p < .001* |
| Fig. 3D, Uracil | 8-10 (f) mice/group | Scheirer-Ray-Hare test followed by targeted Mann-Whitney U test; Bonferroni-Holm corrected | diet: *χ²* (2) = 5.20, *p = 0.0743*  treatment: *χ²* (1) = 22.45, *p < 0.0001*  interaction: χ² (2) = 1.47, *p = 0.4795*  LAD DMF vs. LAD veh: *p = p < .001*  NCD DMF vs. NCD veh: *p = 0.0104*  HFbD DMF vs. HFbD veh: *p = 0.0232* |
| Fig. 4B, Alpha diversity | 9-10 (f) mice/group | Scheirer-Ray-Hare test followed by targeted Mann-Whitney U test; Bonferroni-Holm corrected | diet: *χ²* (2) = 36.63, *p < 0.0001*  treatment: *χ²* (1) = 0.03, *p = 0,8625*  interaction: χ² (2) = 0.46, *p = 0,4976*  HFbD DMF vs. LAD DMF: *p < 0.001*  HFbD DMF vs. NCD DMF: *p < 0.001*  HFbD veh vs. LAD veh:  *p < 0.001*  HFbD veh vs. NCD veh:  *p < 0.001* |
| Fig. 4C, Prevotellamassila | 9-10 (f) mice/group | Scheirer-Ray-Hare test followed by targeted Mann-Whitney U test; Bonferroni-Holm corrected | diet: *χ²* (2) = 26.87, *p < 0.0001*  treatment: *χ²* (1) = 0.74, *p = 0,3897*  interaction: χ² (2) = 0.25, *p = 0,8825*  HFbD DMF vs. LAD DMF: *p = 0.0048*  HFbD DMF vs. NCD DMF: *p = 0.0048*  HFbD veh vs. LAD veh: *p = 0.0025*  HFbD veh vs. NCD veh: *p = 0.0012* |
| Fig. 4C,  *Parabacteriodes* | 9-10 (f) mice/group | Scheirer-Ray-Hare test followed by targeted Mann-Whitney U test; Bonferroni-Holm corrected, | diet: *χ²* (2) = 47.08, *p < 0.0001*  treatment: *χ²* (1) = 0.01, *p = 0.9203*  interaction: χ² (2) = 0.75, *p = 0.6873*  NCD DMF vs. LAD DMF: *p < 0.001*  HFbD DMF vs. LAD DMF: *p < 0.001*  HFbD DMF vs. NCD DMF: *p < 0.001*  NCD veh vs. LAD veh: *p < 0.001*  HFbD veh vs. LAD veh: *p < 0.001*  HFbD veh vs. NCD veh: *p < 0.001* |
| Fig. 4C,  *Acetatifactor* | 9-10 (f) mice/group | Scheirer-Ray-Hare test followed by targeted Mann-Whitney U test; Bonferroni-Holm corrected | diet: *χ²* (2) = 10.20, *p = 0.0061*  *treatment: χ²* (1) = 1.48, *p = 0.2238*  interaction: χ² (2) = 6.64, *p = 0.0362*  NCD DMF vs. LAD DMF: *p = 0.024*  NCD veh vs. LAD veh: *p = 0.0135*  HFbD veh vs. LAD veh: *p = 0.0441*  HFbD DMF vs. HFbD veh: *p = 0.0441* |
| Fig. 5B | 8-10  (3-4f, 5-6m)  mice/group;  excluded, 4 mice found dead (2 DMF and 2 Vehicle) | Scheirer-Ray-Hare test followed by targeted Mann-Whitney U test; Bonferroni-Holm corrected | genotype: *χ²* (1) = 0.90, *p = 0.3428*  treatment: *χ²* (1) = 8.97, p = 0.0027  interaction: χ² (1) = 4.83, *p* = 0.028  *Hca2****^+/+^*** DMF vs. *Hca2****^+/+^*** veh: *p* = 0.0052  *Hca2****^-/-^*** DMF vs. *Hca2****^+/+^*** DMF: *p* = 0.0165  *Hca2^+/+^* veh vs. *Hca2^-/-^* veh*: p* = 0.4044 |
| Fig. 6C, monocytes intermediate | 5-6  (1-4f, 3-5m)  mice/group; excluded, 1 mouse terminated due to eczema (LAD/DMF) | Two-way ANOVA,  with Bonferroni posthoc test | diet: *F* (1,19) = 7.76,  *p* = *0.0118*  treatment: *F* (1,19) = *0.99*,  *p* = *0.3325*  interaction: *F* (1,19) = 0.34,  *p* = *0.5675*  HFbD veh vs. LAD veh:  *p = 0.0493* |
| Fig. 6 – figure supplement 1B, monocytes high | 5-6  (1-4f, 3-5m)  mice/group; excluded, 1 mouse terminated due to eczema (LAD/DMF) | Two – way ANOVA  with Bonferroni posthoc test | diet: *F* (1, 19) = 4.70,  *p* = *0.0431*  treatment: *F* (1,19) = 1.07,  *p* = *0.3142*  interaction: *F* (1,19) = 0.68,  *p* = *0.4181*  HFbD DMF vs. LAD DMF: *p = 0.1047*  HFbD veh vs. LAD veh: *p = 0.6874* |
| Fig. 6 – figure supplement 2A, microglia | 4-6  (1-4f, 3-5m) mice/group | Two-way ANOVA, with Bonferroni posthoc test | diet: F (1, 17) = 8.331, *p = 0.0103*  treatment: F (1, 17) = 0.7878, *p = 0.3872*  interaction: F (1, 17) = 0.8181, *p = 0.3784*  HFbD DMF vs. LAD DMF: *p = 0.0374* |
| Fig. 7B | 4  (1-3f, 1-3m)  mice/group,  50 cells/mouse | One-way ANOVA  with Bonferroni posthoc test | *F* (3,12) = 35.00,  *p* < 0.0001  *Hca2^Fl/Fl^* vs. *Hca2****^nKO^:***  *p = 0.0089*  *Hca2^+/+^* vs. *Hca2****^-/-^:***  *p = <0.0001*  *Hca2^+/+^* vs. *Hca2****^nKO^***  *p = 0.0014, p =* |
| Fig. 7D | 4 (4m) mice per group | Unpaired Student t-test | Hca2^Fl/Fl^ vs Hca2^nKO^: T (6) = 14.59,  *p < 0.0001* |
| Fig. 8B, AUC | 10-13  (5-7f, 4-8m)  mice/group | Scheirer-Ray-Hare test followed by targeted Mann-Whitney U test; Bonferroni-Holm corrected | treatment: *χ²* (1) = 10.35, *p = 0.0013*  *genotype: χ²* (1) = 0.39, *p = 0.5323*  interaction: χ² (1) = 2.30, *p = 0.1294*  *Hca2^Fl/Fl^* DMF vs. *Hca2^Fl/Fl^* veh: *p = 0.0038* |
| Fig. 9C, *Btg2* | 5  (7f, 8m)  mice/group | Two-way ANOVA  with Bonferroni posthoc test | diet: *F* (2,24) = 0.83,  *p = 0.4500*  treatment: *F* (1,24) = *7.37*  *p* = *0.0121*  interaction: *F* (2,24) = 0.87,  *p* = *0.4306* |
| Fig. 9C, *Il1b* | 5  (7f, 8m)  mice/group | Two-way ANOVA  with Bonferroni posthoc test | diet: *F* (2,24) = 0.35,  *p = 0.0356*  treatment: *F* (1,24) = 0.21  *p* = *0.6509*  interaction: *F* (2,24) = 0.42,  *p* = *0.6636* |
| Fig. 10A | 6-8 wells/group | Unpaired Student t-test | HFbD MMF vs. HFbD veh: *T* (14) = 3.99,  *p = 0.0013* |
| Fig. 10B | 5-6 wells/group | One-way ANOVA  with Bonferroni posthoc test | *LAD*  F (2, 15) = 3,795, *p = 0.0464*  CTRL vs. MMF: *p = 0.0447*  *HFbD*  *F* (2, 14) = 5.66, *p = 0.0158*  CTRL vs. PMA: *p = 0.0298*  PMA vs. MMF: *p = 0.0236* |
